# An equity and environmental justice assessment of anti-science actions during the Trump administration

**DOI:** 10.1057/s41271-022-00390-6

**Published:** 2023-02-03

**Authors:** Anita Desikan, Taryn MacKinney, Casey Kalman, Jacob M. Carter, Genna Reed, Gretchen T. Goldman

**Affiliations:** Center for Science and Democracy, Union of Concerned Scientists, Washington, DC USA

**Keywords:** Scientific integrity, Environmental justice, Equity, Attacks on science, Science policy

## Abstract

In the United States, science shapes federal health and safety protections, but political officials can and do politicize federal science and science-based safeguards. Many presidential administrations have politicized science, but under the administration of President Trump, these attacks on science—such as buried research, censored scientists, halted data collection—increased in number to unprecedented levels. Underserved communities bore the brunt of the harms. Such attacks disproportionately harm Black, Indigenous, low-income communities, and communities of color, all of whom have long been burdened by pollution exposure and other stressors. We analyze the effects on underserved communities of the Trump administration’s anti-science environmental and public health policy actions and offer policy recommendations for current and future administrations. Our goal is to strengthen scientific integrity, prioritize health disparity research, and meaningfully engage affected communities in federal rulemaking.

## Key messages

- Political and financial motivations of United States (US) presidential administrations are unfortunately a perennial threat to science-based decisionmaking.
- We analyze how anti-science actions under the Trump administration particularly impacted Black, Indigenous, low-income communities, and communities of color.
- Current and future administrations should create policy infrastructure that promotes independent science and ensures decisions have equitable benefits.

## Introduction

The US Congress, by enacting legislation, has charged federal agencies with protecting public health and the environment [1, 2]. Many such safeguards, including air pollution standards and restrictions on toxic chemicals, rely on scientific evidence. In practice, federal agencies sometimes fall short of fully executing their congressionally mandated duties to protect public health and the environment. There may be a variety of causes, but one contributing factor is willingness of some government officials to sideline science for political, financial, or ideological reasons. Not surprisingly, these actions often align with presidential administration priorities. These losses of scientific integrity may weaken health protections in communities across the US, especially in underserved communities.

Underserved communities (Black, Indigenous, and low-income communities, and communities of color) are those faced with significant barriers to accessing the benefits associated with environmental and public health protections, and often experience the brunt of harms when policymakers sideline science. This, in turn, exacerbates long-standing health inequities. Since at least the 1980s, a growing body of research has shown that members of underserved communities face disproportionately high exposure to pollution and other stressors [3–8]. Residents of underserved communities are exposed to greater health hazards in their homes, workplaces, and neighborhoods than are residents from whiter and more affluent communities. The hazards relate to long-standing inequities and systemic racism, such as residential segregation due to ‘redlining’ practices in which governments marked up neighborhoods on maps and gave lower grades in red to places where they expected property values to decrease, often in areas with Black homeowners [9]. Underserved communities are more likely to be located near sources of environmental hazards such as sewage systems, mines, landfills, industrial facilities, major roads, and fossil fuel extraction operations [6]. A groundbreaking study conducted in 1987 found that race was the most potent variable for predicting location of commercial hazardous waste facilities in the US. [3]. Evidence continues to build, making it ever clearer that underserved communities face disproportionately higher exposure to environmental harms [6, 7], that may cause cardiovascular and respiratory diseases, cancer, and death [8, 10].

For decades, environmental justice advocates, scientists, and members of underserved communities have advocated for policymakers to use a framework for analyzing environmental justice centered on a right of all individuals to be protected from environmental degradation; adopt a public health strategy based on scientific evidence to identify threats to underserved communities, particularly before these threats result in harm; shift the burden of scientific proof to polluters to show that their activities are not resulting in harm to underserved communities; and target resources toward communities experiencing disproportionate adverse health effects from environmental hazards [4, 5].

Implicit in this framework is the role of robust and independent science to identify health disparities and for policymakers to rely on the best available science to carry out policy actions equitably. Science-based and science-informed decision making can help counteract implicit bias and systemic racism in policy decisions, and, in turn, help protect underserved communities from these health threats [11–13]. When policymakers choose to sideline science when making decisions, they undermine the pressing need to address disproportionate health outcomes in underserved communities. These communities face cumulative effects from a range of unjust policy decisions (such as job instability, unfair wages, less access to government aid, and inadequate healthcare coverage) that exacerbate the harms and make recovery from them more difficult [7, 14]. For some political officials, undermining science is a powerful tactic to shape regulation. Outside groups with financial incentives, such as corporations, have long established a playbook on how to do so [15, 16]. Officials can bury research, censor scientists, cut off funding for scientific research conducted by agency scientists or otherwise funded by agencies, or stop data collection [17–19]. How officials handle science and pressures from outside groups indicates whether a governmental system values and incorporates science-based decision making and whether underserved communities experience health burdens as a result of exposures to environmental health threats.

This connection is particularly prominent at science-based federal agencies that employ thousands of scientists to carry out robust and independent data collection and analyses. These same agencies employ political appointees in high-level positions who may sideline science. Within the federal government, this tension is usually described in reference to scientific integrity, a set of principles, guidelines, and policy documents adopted by federal agencies to ensure that rigorous scientific research is free from politically motivated suppression or distortion [11, 13, 20–23]. Since 2010, federal agencies have worked to establish scientific integrity policies, stronger or weaker ones depending on the agency [24]. The administration of President Biden, through its Office of Science and Technology Policy (OSTP), has worked to harmonize and institutionalize stronger scientific integrity policies across agencies and to explicitly include in them commitments to equitable workplaces and prioritization of science-based decisions that affect underserved communities [11–13].

While every administration since at least the 1950s has sidelined science to advance a political agenda [17], the Trump administration’s attacks on science were unprecedented in frequency [16, 18, 19, 22, 23]. And they often hindered progress on environmental justice [25–31]. In the context of equity and environmental justice, the clearest examples of the Trump administration’s anti-science actions occurred at the Environmental Protection Agency (EPA) with attempts to defund and reduce staff and undermine the agency’s science-based policymaking processes [16, 27–31]. Previous research suggests that this led to a culture of fear and censorship among thousands of EPA scientists. Federally employed scientists surveyed in 2018 across 16 agencies, including at the EPA, reported a diminished focus on equity and environment justice [26]. At least through 2020, however, research also suggests that environmental justice had been traditionally devalued, underfunded, and marginalized by presidential administrations for decades, especially within the EPA’s environmental justice office [29]. Thus, this history of repeated sidelining of environmental justice across administrations provides important context for evaluating effects of further erosion of science and equity at an unprecedented pace during the Trump administration.

The relationship between the Trump administration’s diminution of scientific integrity and related harms on underserved communities has not yet been discussed in the literature. Several universities, law firms, newspapers, and non-profit organizations tracked and documented the Trump administration’s anti-science actions and deregulatory rulemakings in real time, but none of these efforts examined the effect of the actions on underserved communities [18, 19].

**Fig. 1:**
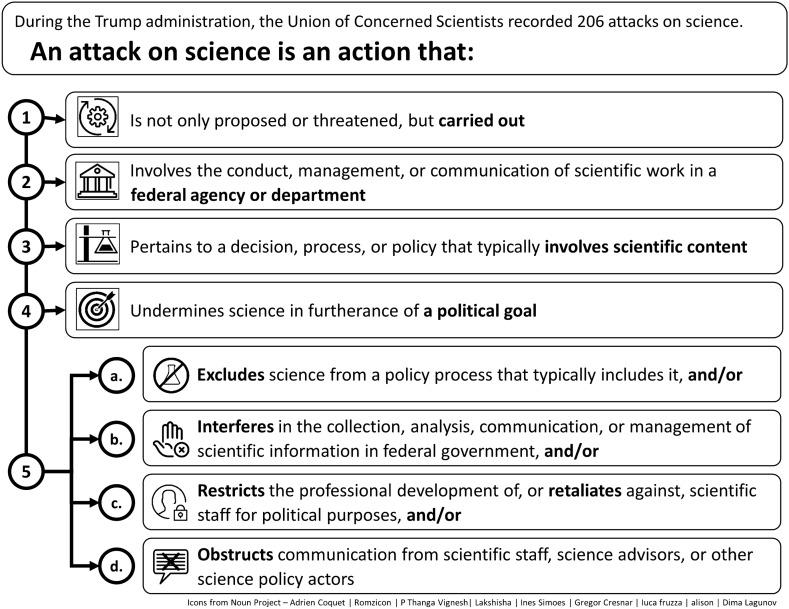
The Union of Concerned Scientists' criteria to characterize an attack on science

**Fig. 2:**
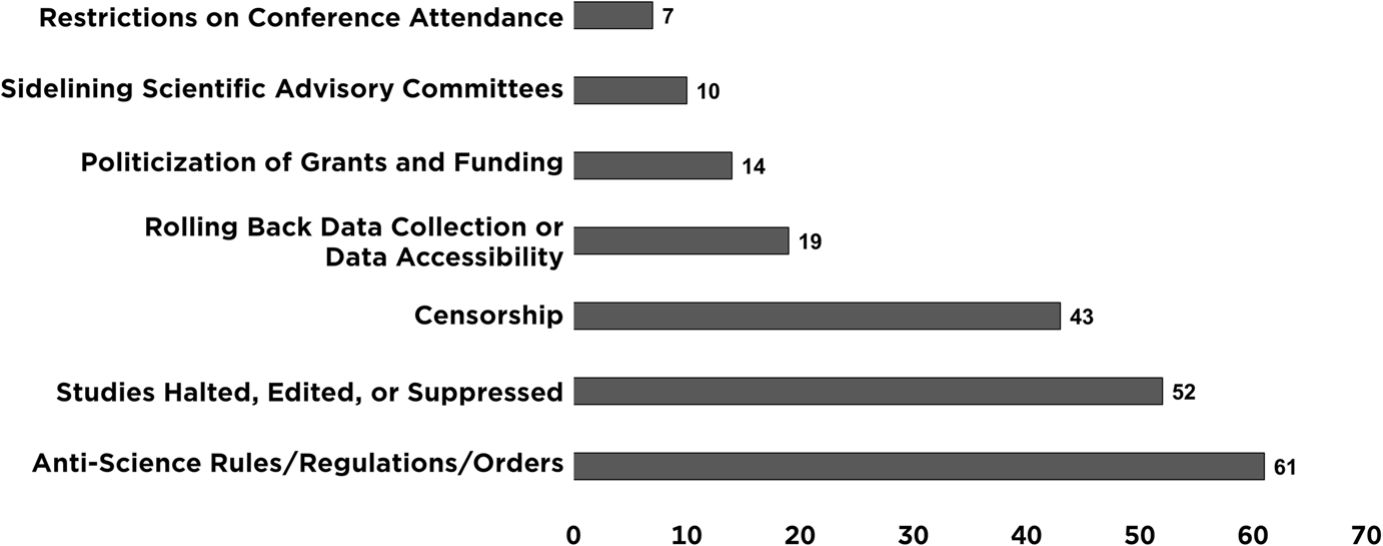
The Union of Concerned Scientists documented 206 attacks on science during the Trump administration

To understand the role of science in decision making and environmental justice, we examined the effects of the Trump administration’s anti-science actions using two approaches. First, to understand the scope, breadth, and frequency, we analyzed the data and documented incidents of attacks on science by the Trump administration (Figs. 1, 2). We also analyzed the data on attacks on science that we determined had an effect on underserved communities. Second, to contextualize how these attacks on science undermined the science policy process, particularly for underserved communities, we examined a set of equity and environmental justice case studies and focused on how policy gaps and breaches of agency norms made the agencies vulnerable to losses of scientific integrity. Finally, we used data from the attacks on science that had effects on underserved communities to outline how current and future administrations can bolster or create policy infrastructure with the dual purposes of ensuring independent science and placing environmental justice at the center.

## Methods

We collected information to allow us to analyze patterns in how the Trump administration attacked science. To examine the effects on underserved communities, we conducted a sub-analysis on anti-science actions that had an equity component. After examining publicly available sources from January 2017 to November 2022, we created an online database that tracked instances of when the Trump administration attacked science.

The publicly available information came from agency websites, the federal register, newspaper articles, academic journals, congressional websites, agency inspector general reports, and Government Accountability Office (GAO) reports. To assess whether an action undermined science with intention to further a political goal, we often relied on documents (emails and unpublished reports) that we obtained through requests under the US Freedom of Information Act or other ‘sunshine laws’ that require governments to make staff communications, data, documents, and other information publicly available upon request. As details of the Trump administration’s treatment of science continued to emerge in 2021 and 2022 from responses to our document requests and interviews with former government staff, we continued tracking these attacks on science until November 2022.

We defined our study time period to coincide with presence of the Trump administration. It started on January 21, 2017 (his first full day in office) to January 20, 2021 (the day his term ended). We used rigorous criteria to assess whether an action carried out by the Trump administration constituted an attack on science (Fig. 1). We excluded research misconduct cases that appeared not to be politically motivated; that is, we determined they did not constitute attacks on science. We also excluded instances of when President Trump simply communicated something that was not science-based, or did not adequately capture the best available science, because these did not amount to decisions about policy. When assessing policy decisions that were not in line with the best available science, we only considered them an attack on science if language in the law (statutes) required use of science or if the agency had established a science-based best practice or norm in the past. Based on this definition we identified 206 attacks on science.

We analyzed these data using a descriptive analysis approach. We used measures of frequency to characterize the trends and patterns that characterized the administration's anti-science actions. When we identified an attack, we documented the agency or agencies involved, the year of the attack and the trend in substance, the type of science-based issue at stake, and how the perpetrators carried out the attack [32]. A single agency or multiple agencies could be associated with one recorded attack on science. The agencies involved could be either the perpetrator or the target of the attack. When documents listed the White House (Presidential headquarters) or the Executive Office of the President of the US as an involved agency, it was always as a perpetrator. We evaluated the type of science-based issue at stake using six issue areas: climate change, COVID-19, endangered species, environmental, equity, and public health. These categories were not mutually exclusive (some attacks were associated with more than one issue area) and not all recorded attacks on science involved one of these six issue areas. Thirty-three attacks did not involve climate change, COVID-19, endangered species, environmental, equity, and public health. When assessing the means by which perpetrators carried out an attack (Fig. 2), we used seven mutually exclusive categories and all attacks fell into just one of these: anti-science rules/regulations/orders; studies halted, edited, or suppressed; censorship; rolling back data collection or data accessibility; politicization of grants and funding; sidelining scientific advisory committees; or restrictions on conference attendance.

## How the Trump administration attacked science

During the Trump administration, we at the Union of Concerned Scientists documented 206 attacks on science, a total far exceeding those documented during the administrations of George W. Bush (98) and Barack Obama (19) [32]. Commonly but not always, an attack violated an agency or department policy or standard practice, ran counter to an agency or department’s mission statement, or was an action taken by a political appointee for political gain (Fig. 1). A systematic recording and documenting of the Trump administration’s attacks on science using the same criteria represents an unusual analysis. We are aware of only one other research group that has used a similar set of methodologies to analyze attacks on science [18].

Attacks on science commonly fell into the six issue areas we chose, with the highest counts as follows: environmental (79), public health (58), climate change (34), equity (27), COVID-19 (29), and endangered species (20). Sixty-nine attacks encompassed one or more issue areas, with the three most prevalent combinations being public health and environmental (34), endangered species and environmental (8), and public health and equity (6). The combination of public health and environmental mostly involved chemical safety issues. Of the 206 attacks, 33 did not fit into any of these 6 issue areas, and involved administrative procedures, communication with the media, data and research accessibility, scientific advisory committees, scientific grants, or other scientific topic areas (e.g., energy, economics, or political science). We also categorized all 206 attacks by type—the three most common were political appointees issuing anti-science rules (61), suppressing studies (52), or censoring federal scientists (43) (Fig. 2). Forty agencies were involved in the 206 attacks. The five most often involved included the EPA (64), White House (31), Department of the Interior (DOI) (30), Department of Health and Human Services (HHS) (25), and Centers for Disease Control and Prevention (CDC) (22).

Over time, the Trump administration shifted some patterns of attack on science:

- In 2017, the Trump administration carried out 54 attacks that often had the effect of stemming the flow of agency science to the public, such as through the censorship of climate science. Some of the most explicit attacks on underserved communities also occurred in this year. For instance, the Trump administration initially barred release of an economic study by HHS that showed refugees contributed $63 billion more to government revenue than they used in public services. Senior White House officials wrote in an email that they questioned the “assumptions used to produce this report.” They issued a summary that described only the costs associated with refugees, never the full report [33]. Soon after, the Trump administration adopted a historically low target (45,000) for the number of refugees to allow into the country over the next fiscal year [34].
- In 2018, the 26 attacks continued several trends observed in the prior year, like preventing scientists from attending conferences, suppressing scientific reports, and restricting public access to data and research. The Trump administration first started to issue finalized rules and regulations that were out of line with the best available scientific information.
- In 2019, the 46 attacks were on a wide variety of topic areas. Most of the attacks on endangered species (9 out of 20) occurred during this year.
- In 2020, the 55 attacks mostly dealt with the COVID-19 pandemic and the CDC was an especially frequent target. With two exceptions, all 22 attacks on science involving the CDC occurred during the first year of the COVID-19 pandemic and involved this disease.
- In 2021, 10 out of 22 attacks occurred before the end of the Trump presidency on January 20, 2021 and mostly involved the issuing of anti-science rules or regulations. Attacks reported after this date in 2021 or in 2022 (3) surfaced due to investigatory actions by the federal government, Congress, and the media.

We took a closer look at the 27 attacks on science that had an equity component—attacks that likely affected underserved communities. Of the 14 agencies involved in these, the top 5 included HHS (6), DOI (4), EPA (3), Census Bureau (3), White House (2), Department of Justice (DOJ) (2), Occupational Safety and Health Administration (OSHA) (2), Department of Homeland Security (DHS) (2), and CDC (2). The greatest number of attacks fell into three of the categories: anti-science rules, regulations, and orders (10), studies halted, edited, or suppressed (7), and rolling back data collection or data accessibility (6). We determined that perpetrators of some of these attacks clearly meant to undermine science to disenfranchise underserved communities.

## Case study and evidence

Policymaking occurs in stages—identifying a problem, creating a policy with public input, then implementing and enforcing it. In practice, policymakers sometimes skip stages or complete them out of order [35]. Nevertheless, these stages provide a useful framework for conceptualizing policymaking and understanding how policymakers apply science throughout the process. Below, we outline losses of scientific integrity in three policymaking phases, using case studies from the Trump administration to highlight how the administration sidelined science to the detriment of underserved communities.

### Identifying a problem

Federal agencies collect scientific data and conduct scientific research to identify, monitor, and address public health and safety risks, evaluate policy effectiveness, and identify inequities in public services. When limited or no data are available, science agencies may not take policy action. Thus, the federal government’s ability to identify and address environmental justice issues relies on robust research and data collection.

In August 2017, Hurricane Harvey damaged over 40 petrochemical facilities around a City, Houston, in the State of Texas. These facilities released pollutants that quickly concentrated in underserved communities [14, 36, 37] and exacerbated disparities in industrial pollution exposure in them [38, 39]. National Aeronautics and Space Administration (NASA) scientists offered the EPA and the Texas Commission on Environmental Quality (TCEQ) cutting-edge technology to measure air pollutant levels in and around Houston communities. The EPA and TCEQ blocked the effort, arguing that the data would cause “confusion” and introduce “conflicting” data [40].

By preventing comprehensive data collection in an emergency, the administration deliberately restricted the body of available data on pollutants known to cause adverse health effects. Consequently, people affected by the crisis could not make decisions based on the best available science to keep themselves and their loved ones safe. Decision makers could have used such data to assess the health effects of the crisis, inform emergency response, and shape policies to better protect the public—and especially underserved communities—from disasters in future.

### Creating a policy with public input

The US Government is beholden to its people, and public participation in evidence-based policymaking is central to the Constitution’s stated vision of self-governance. Public participation (public comments, listening sessions, and hearings) gives people a voice in policy decisions [41, 42]. For decades, government officials have failed to include underserved communities at the science policymaking table or failed to meaningfully incorporate lived experience or Tribal ecological knowledge in decisions, despite underserved communities often having been the constituents most affected by government decisions [43, 44].

In April 2017, DOI leaders began to identify national monument sites from which to strip federal protections and opened this review to a public comment process [45]. One such national monument was Bears Ears, the first created at the request of and with input from Indigenous governments [46]. In a recorded webcast (that we obtained through a Freedom of Information Act request) DOI officials trained staff members how to evaluate public comments. The senior official leading the training appeared to dismiss the process, saying that, “barring a surprise, there is no new information that’s going to be submitted” in public comments. Instead of weighing evidence and public opinion, officials emphasized the value of the land to ranching and energy development. This led to weakened protections for Indigenous lands, fewer tourism opportunities for the public, and threatened archaeological sites [47]. Resource extraction industries frequently choose sites on Indigenous lands, leading to health disparities [48]. Oil pipelines can contaminate Indigenous food sources, and abandoned uranium mines can increase the risk of kidney disease and hypertension in Indigenous residents [49, 50]. By selecting only evidence that favored the needs of extractive industries and disregarding feedback from public commenters, several of them from Indigenous communities, the administration undermined a central tenet of democratic governance—public participation. It made decisions that neither represented public interests nor served to benefit environmental quality or public health.

### Implementing and enforcing a policy

Because alleviating health inequities depends upon strong science-based protections, the implementation and enforcement of these policies play major roles in the health and safety of communities.

In November 2020, the EPA issued a final rule eliminating a 1995 once in, always in guidance for major sources under the Clean Air Act [51]. Before its remission, the policy required major polluters, including many petrochemical manufacturers, once classified as “major sources” to abide by tight emission standards and keep the “major” classification even after making emissions reductions in the short term. The Trump administration’s policy change allowed some industrial polluters to reclassify themselves and avoid a requirement in the Clean Air Act to use maximum achievable control technology, the gold standard for reducing industrial emissions of carcinogenic pollutants such as benzene. A study examined the potential effects of this policy action by the Trump administration and found that up to 70% of major polluters could shift to a less stringent standard, resulting in the emission of up to 35,000 additional tons per year of hazardous air pollutants [52]. Hazardous air pollutants are known or suspected to cause cancer or other serious health effects [53].

As a result of long-standing, systemic inequities and resulting zoning and land use laws industrial sources of hazardous air pollutants are more likely to locate their facilities near communities with higher proportions of low-income individuals and people of color [6]. Exposure to these facilities’ toxic emissions can result in asthma, heart attacks, cancer, and premature death [8, 10]. By dismantling compliance and enforcement safeguards from industrial pollution, the Trump administration put underserved communities at greater risk of detrimental health outcomes.

## Policy recommendations

Decisions informed by science are especially important to address the health inequities faced by underserved communities. To strengthen scientific integrity and incorporate equity and environmental justice into decision making, federal agencies can implement several recommendations [20, 21, 54]. Agencies should also consider implementing preventative measures that guard against interference from future attacks on science.

### Research and data collection

#### Consider cumulative impacts

Agencies should carry out cumulative impact analyses to provide a holistic view of health and safety risks faced by communities and adequately account for all sources of pollution, including those from fugitive releases from refineries, chemical plants, and other industrial facilities during startups, shutdowns, and malfunctions [55].

#### Prioritize research on health disparities

Prioritize grant solicitations for, and otherwise encourage research on efforts that can highlight health disparities in underserved communities.

#### Disaggregate data

Agencies should disaggregate and make publicly available to the greatest extent possible, environmental data on human health risks and exposures by race, ethnicity, gender, age, income, and geographic location [12].

### Community participation

#### Improve proactive outreach efforts

Agencies should amend existing practices on public participation to require outreach to identify and meaningfully engage with communities, including by actively working to address barriers to participation (language barriers or Internet inaccessibility).

#### Research and implement better public engagement strategies

Agencies should carry out research to identity effective strategies for engaging the public during rulemaking, particularly for communities most affected by proposed rules.

#### Solicit public input effectively

Agencies should ensure that the public can comment early and effectively in the rulemaking process to promote a deliberative model of public engagement that encourages two-way dialogue between agencies and the public [42].

### Implementation and enforcement

#### Strengthen the Environmental Justice Executive Order (EO 12898)

To strengthen the implementation of Executive Order 12898, issued in 1994 by President Bill Clinton to focus federal attention on the environmental and human health effects in underserved communities, each federal agency, to the maximum extent permitted by law, should include diverse segments of the population in epidemiological and clinical studies, identify multiple and cumulative exposures, and actively encourage and solicit community-based science and Tribal ecological knowledge [56].

#### Prioritize rapid risk mitigation for underserved communities

When the EPA’s Integrated Risk Information System—a database of chemical safety assessments—updates a risk value for a chemical and it poses an adverse public health risk, the EPA should notify communities and prioritize enforcement and cleanup efforts. EPA should intervene most rapidly where facilities near underserved communities emit that contaminant.

#### Build a robust inspection and compliance workforce

Agencies must fill all safety inspector positions and expand the workforce responsible for health and safety investigations.

#### Fund enforcement activities

Science-based agencies have limited capacity for enforcement of environmental regulations due to inadequate funding. This limits their ability to enforce, ensure compliance with, and impose meaningful penalties for violations of health-protecting environmental laws. Agencies must request and advocate for well-funded budgets to implement these regulatory and enforcement programs.

## Conclusion

By examining how actions taken under the Trump administration undermined scientific integrity and harmed underserved communities, we can better understand how current and future administrations can safeguard science and its role in protecting underserved communities. Science conducted and funded by the federal government represents a powerful tool for the public good, particularly in pollution-burdened communities. It is imperative that federal scientists do their jobs without political interference. To protect the health and safety of the country’s most disenfranchised, government decision makers must safeguard the scientific process and ensure that science and equity inform policy at every stage. Further, the examples provided in this study show the breadth of ways in which sidelining science in decisionmaking can take shape, with lessons and solutions that can be applied at other levels of government in the US as well as in other countries facing similar threats.

## Data Availability

The attacks on science dataset that support the findings of this study are available in the Dataverse repository. https://dataverse.harvard.edu/dataset.xhtml?persistentId=doi:10.7910/DVN/IFVLOW
